# Improved pressure contour analysis for estimating cardiac stroke volume using pulse wave velocity measurement

**DOI:** 10.1186/s12938-017-0341-z

**Published:** 2017-04-24

**Authors:** Shun Kamoi, Christopher Pretty, Joel Balmer, Shaun Davidson, Antoine Pironet, Thomas Desaive, Geoffrey M. Shaw, J. Geoffrey Chase

**Affiliations:** 1 Department of Mechanical Engineering, University of Canterbury, Christchurch, New Zealand; 2 GIGA Cardiovascular Science, University of Liege, Liege, Belgium; 3 Intensive Care Unit, Christchurch Hospital, Christchurch, New Zealand

**Keywords:** Pressure contour analysis, Hemodynamic monitor, Stroke volume, Pulse wave velocity, Reservoir–wave pressure, Water hammer, Cardiovascular system, Physiological modelling, Windkessel model, Intensive care

## Abstract

**Background:**

Pressure contour analysis is commonly used to estimate cardiac performance for patients suffering from cardiovascular dysfunction in the intensive care unit. However, the existing techniques for continuous estimation of stroke volume (SV) from pressure measurement can be unreliable during hemodynamic instability, which is inevitable for patients requiring significant treatment. For this reason, pressure contour methods must be improved to capture changes in vascular properties and thus provide accurate conversion from pressure to flow.

**Methods:**

This paper presents a novel pressure contour method utilizing pulse wave velocity (PWV) measurement to capture vascular properties. A three-element Windkessel model combined with the reservoir–wave concept are used to decompose the pressure contour into components related to storage and flow. The model parameters are identified beat-to-beat from the water-hammer equation using measured PWV, wave component of the pressure, and an estimate of subject-specific aortic dimension. SV is then calculated by converting pressure to flow using identified model parameters. The accuracy of this novel method is investigated using data from porcine experiments (N = 4 Pietrain pigs, 20–24.5 kg), where hemodynamic properties were significantly altered using dobutamine, fluid administration, and mechanical ventilation. In the experiment, left ventricular volume was measured using admittance catheter, and aortic pressure waveforms were measured at two locations, the aortic arch and abdominal aorta.

**Results:**

Bland–Altman analysis comparing gold-standard SV measured by the admittance catheter and estimated SV from the novel method showed average limits of agreement of ±26% across significant hemodynamic alterations. This result shows the method is capable of estimating clinically acceptable absolute SV values according to Critchely and Critchely.

**Conclusion:**

The novel pressure contour method presented can accurately estimate and track SV even when hemodynamic properties are significantly altered. Integrating PWV measurements into pressure contour analysis improves identification of beat-to-beat changes in Windkessel model parameters, and thus, provides accurate estimate of blood flow from measured pressure contour. The method has great potential for overcoming weaknesses associated with current pressure contour methods for estimating SV.

## Background

Stroke volume (SV) is an important physiological parameter for diagnosing and treating patients suffering from cardiovascular dysfunctions [1–4]. Accurately tracking changes in SV provides an in-depth picture of cardiovascular condition and response to therapy, thus enabling more optimal care [5, 6]. Although SV measurements are increasingly seen as essential for correct clinical decisions, accurate continuous SV measurement requires highly invasive instrumentation, such as inserting an admittance catheter into the ventricle and is thus not clinically feasible.

At present, SV is continuously measured indirectly using continuous cardiac output monitors in the intensive care unit (ICU) [7, 8]. Non-invasive methods, such as esophageal Doppler and bio-impedance exist, but can be operator-dependent [9] and inaccurate due to the signal quality [10]. Moderately invasive methods, such as pressure contour analysis have been shown to be unreliable during hemodynamic instability [11]. The error comes from calibration methods involving use of fixed arterial properties, such as peripheral resistance, compliance, and vascular impedance [12], which certainly change as patient condition evolves and due to clinical interventions.

Inaccurate surrogate measures of SV could lead to misdiagnosis, incorrect clinical treatment/decisions, and/or misinterpretation of patient response to therapy. In the ICU, changes in the hemodynamic state are expected, and assuming constant hemodynamic parameters may not be suitable in many situations, such as fluid resuscitation [13] and/or inotrope therapy [14]. Therefore, there is a need for accurate and robust methods to estimate SV that are reliable even when hemodynamic properties are evolving rapidly.

This paper presents a novel pressure contour method for estimating SV. A three-element Windkessel model [15] combined with the reservoir–wave concept [16] are used to analyse the pressure contour. Windkessel parameters are identified using pressure and pulse wave velocity (PWV) measurements using a calibrated arterial diameter. Identified parameters are used to calculate flow from pressure measurements and SV is then calculated by integrating the estimated flow.

The distinct difference between the proposed method and the traditional methods [17, 18] is that PWV measurements are used to improve the capacity of pressure contour analysis. Furthermore, reservoir–wave separation was performed on the pressure waveform to accurately capture the changes in Windkessel parameters [19]. The separation provides more accurate information on which Windkessel parameter is responsible for the changes in the shape of pressure waveform.

Pressure contour analysis has traditionally been purely based on the morphology of the pressure waveform alone [20]. Consequently, the weaknesses associated with monitoring SV were always found during hemodynamic instability [21], which is the exact point where monitoring patients SV becomes essential. To overcome the limited information achievable from a single measurement, the presented method couples pressure and PWV measurements to improve the analysis of circulatory system. To investigate the accuracy of this new method, Bland–Altman plots are presented for data from porcine experiments, where beat-to-beat SV is estimated and compared against SV measured directly from an admittance catheter.

## Methods

### Porcine experiments

All experimental procedures, protocols and the use of data in this study were reviewed and approved by the Ethics Committee of the University of Liege Medical Faculty.

Experiments were performed on four healthy pigs weighing between 20 and 24.5 kg and a total of 13,409 heart beats were recorded. The pigs were premedicated with ketamine (20 mg/kg) and diazepam (1 mg/kg). Anesthesia was induced and maintained by a continuous infusion of sufentanil (0.5 µg/kg/h) and sodium pentobarbital (3 mg/kg). The pigs were intubated via tracheotomy and ventilated using a GE Engstrom Carestation ventilator (GE healthcare, Chicago, United States).

Cardiovascular measurements were continuously recorded using Notocord-hem software (Notocord, Croissy-sur-Seine, France). Left ventricular pressures and volumes were measured using 7F micromanometer-tipped admittance catheters (Transonic Scisense Inc., Ontario, Canada) inserted into the ventricles through the right carotid artery. Central pressure waveform measurements were captured at the aortic arch and abdominal aorta with 7F pressure catheters (Transonic Scisense Inc., Ontario, Canada). Catheters for central pressure waveform measurements were inserted into the aortic arch through left carotid artery and the abdominal aorta through the femoral artery, respectively. All cardiovascular and respiratory data were recorded with the chest closed and sampled at 250 Hz. The analysis in this study was performed using Matlab (version 2015a, The Mathworks, Natick, Massachusetts, USA).

### Experimental protocol

The experiment comprised three different interventions intended to induce hemodynamic variations: (1) volume expansion by saline solution; (2) continuous infusion of the inotrope dobutamine; and (3) step-wise changes in positive end expiratory pressure (PEEP) recruitment manoeuvres (RM). For each pig the experiment started with administration of five rapid saline bolus of 180 ml up to a total of 900 ml, except for Pig 4 where a total of 720 ml of saline solution was given. After fluid administration, the pigs received a continuous infusion of dobutamine at a rate of either 2.5 or 5 µg/kg/min. In Pigs 3 and 4, further administration of a 180 ml rapid bolus were given while continuing dobutamine infusion. The summary of interventions made for each pig are given in Table 1, and provide a wide range of induced hemodynamic changes.

**Table 1:** Summary of interventions made for each pig in the experiment

Pig no.	Volume expansion					Dobutamine (µg/kg/min)		Dobutamine + volume expansion
	180 ml	360 ml	540 ml	720 ml	900 ml	2.5	5	180 ml
1	✓	✓	✓	✓	✓	×	✓	×
2	✓	✓	✓	✓	✓	×	✓	×
3	✓	✓	✓	✓	×	✓	×	✓
4	✓	✓	✓	✓	✓	×	✓	✓

**Fig. 1:**
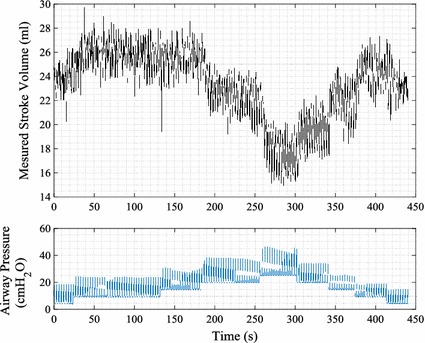
Example of relationship between directly measured SV and PEEP from an experiment. *Top panel* measured SV from left ventricular admittance signal. *Bottom panel* measured airway pressure from mechanical ventilator

Between each intervention, a RM was performed to induce changes in cardiac preload. An increased PEEP results in a smaller pressure gradient between the peripheral veins and the right atrium and decreases systemic venous return [22]. In addition, pulmonary circulation resistance is increased, and as a consequence, left ventricular SV is reduced [23]. RMs involved increasing PEEP with increments of 5 cm H_2_O to a maximum of 20–25 cm H_2_O and then reducing PEEP back to the original PEEP level of 5 cm H_2_O in a step-wise manner. An example of the relationship between SV and PEEP are shown in Fig. 1.

### Pulse wave velocity measurements

**Fig. 2:**
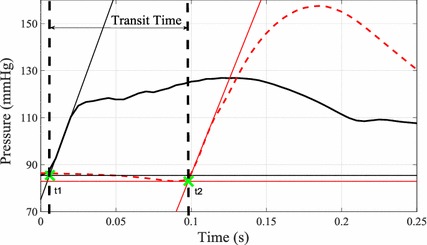
Example of measured aortic arch (*black*) and abdominal aorta (*dashed red*) pressure waveforms. *Thin lines* represent tangent line along maximum pressure gradient and* horizontal lines* along minimum pressure point for each of the waveform. The *green crosses* represent the identified ‘foot’ of the pressure waveforms at times t1 and t2 for aortic arch and abdominal pressure, respectively. Transit time can be seen as the difference (t2–t1) between these time points

The velocity of the pressure wave along the aorta was calculated using the aortic arc and abdominal pressure waveform measurements. The transit time of the pressure wave was measured by locating the ‘foot’ of the systolic rise in each measurement. In this study, the ‘foot’ of the pressure was identified as intersection of the tangent line along the maximum systolic pressure gradient and the horizontal line along the minimum pressure point. Example of the identified ‘foot’ in a single heart beat is shown in Fig. 2.

The distance between the two pressure catheters was measured on the body surface by approximating the catheter locations. This method may introduce error in the absolute value of PWV, but as the catheter locations were fixed throughout each experiment, PWV trends for a given pig were unaffected.

### Detection of end systolic point

**Fig. 3:**
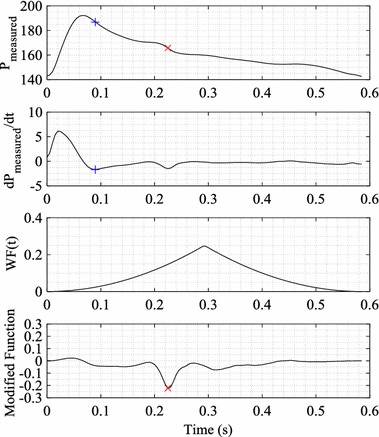
Detected maximum negative gradient point (*blue +*) and detected end systolic point using the modified function (*red x*) on a single beat pressure waveform. *Top panel* measured pressure waveform having multiple descending inflection points. *Second panel* pressure gradient *dP*_*measured*_ */dt* and detected global minimum, shown as *blue cross*. *Third panel* weight function WF(t) applied to *dP*_*measured*_ */dt*. *Bottom panel* modified function showing product of *dP*_*measured*_ */dt* and WF(t), where the *red cross* shows the identified location of the end systolic point

This analysis requires the maximum negative gradient of the pressure waveform per beat, characterised by end systolic point (ESP) [24]. However, this condition is not robust enough for typically measured pressure waveforms, which can have multiple inflection points and thus multiple minima in the dP/dt waveform. An example is shown in Fig. 3.

Figure 3 shows multiple local and global minimum gradient points. In this specific case, the correct location of the ESP would be the second minimum gradient point and not the global minimum point. To avoid false detection of the ESP, which is required for calculating SV, a generic Weight Function (WF) was applied to the gradient of the pressure curve.

1 $$WF\left( t \right) = \left( {0.5 - \left| {0.5 - \frac{HR.t}{60}} \right|} \right)^{2}$$

2 $$ESP = min\left( {\frac{{dP_{measured} }}{dt}WF(t)} \right)$$

where HR is the heart rate.

This approach applies a greater weighting to points near the midpoint of the cardiac cycle and thus, enhances minima in the expected ESP location. This procedure is illustrated in Fig. 3.

### Pressure contour analysis

A measured continuous abdominal pressure waveforms was used to estimate SV in this investigation. The continuous waveforms were first split into individual heart beats for the beat-to-beat pressure contour analysis. For each beat, a reservoir–wave separation was applied and the wave component of the pressure waveform was identified. Aortic characteristic impedance was then calculated by solving the water-hammer equation. Finally, the wave component of the pressure is used with the calculated value of aortic characteristic impedance to estimate beat-to-beat SV.

### Reservoir–wave separation

The time-varying reservoir pressure proposed by Wang et al. [25] is used to analyse the pressure contour. This model interprets the measured pressure as a sum of two pressure components, reservoir pressure *P*_*res*_, and excess pressure *P*_*ex*_.

3 $$P_{measured} \left( t \right) = P_{res} (t)_{{}} + P_{ex} (t)$$

*P*_*res*_ represents the energy stored and released by the aortic compliance, and *P*_*ex*_ is the wave component of the pressure waveform and represents the excess amount of work provided by the ventricle to induce flow in the aorta. The time dependent *P*_*res*_ *(t)* can be expressed as a function of volumetric compliance *C*_*v*_ (=*dV/dP*_*res*_), and changes in aortic compartment volume with respect to time (*dV/dt*), yielding:

4 $$\frac{{dP_{res} (t)}}{dt} = \frac{{dP_{res} (t)}}{dV}\frac{dV}{dt} = \frac{1}{{C_{V} }} \left( {Q_{in} \left( t \right) - Q_{out} \left( t \right)} \right)$$

where *Q*_*in*_ *(t)* and *Q*_*out*_ *(t)* are flow entering aortic compartment from the left ventricle and flow leaving the aortic compartment, respectively. The theory also describes the proportionality between each pressure component and flow dynamics in the aorta:

5 $$P_{res} \left( t \right) - P_{cvp} = RQ_{out} (t)$$

6 $$P_{ex} \left( t \right) = Z_{ao} Q_{in} (t)$$

where *R, Z*_*ao*_ and *P*_*cvp*_ are peripheral resistance, aortic characteristic impedance and central venous pressure, respectively. In this analysis, *P*_*cvp*_ was assumed 8 mmHg for all the pigs as a typical value that could also be measured. Substituting Eqs. (5) and (6) into Eq. (4), the differential equation for reservoir pressure can be written.

7 $$\frac{{dP_{res} (t)}}{dt} = \frac{{P_{ex} (t)}}{{Z_{ao} C_{V} }} - \frac{{P_{res} \left( t \right) - P_{cvp} }}{{RC_{V} }}$$

Assuming that aortic volumetric compliance *C*_*v*_ can be written in the form *C*_*v*_ = *C*_*A*_ *L*_*ao*_, where *C*_*A*_ and *L*_*ao*_ are compliance per unit length of aorta and aortic length, respectively, and PWV can be described by aortic characteristic impedance and compliance per unit length [26], Eq. (7) can be rewritten;

8 $$PWV = 1/Z_{ao} C_{A}$$

9 $$\frac{{dP_{res} (t)}}{dt} = \frac{PWV}{{L_{ao} }}P_{ex} (t) - \frac{{P_{res} \left( t \right) - P_{cvp} }}{{RC_{V} }}$$

By substituting Eq. (3) into Eq. (9) and applying the initial condition that *P*_*res*_ *(0)* = *P*_*measured*_ *(0)* at the beginning of a heartbeat, Eq. (9) can be solved for *P*_*res*_ *(t)* over one cardiac cycle:

10 $$P_{res} \left( t \right) = e^{{ - \left( {\alpha + \beta } \right)t}} \left( {\mathop \int \limits_{0}^{t} e^{{\left( {\alpha + \beta } \right)t^{'} }} \left( {\alpha P_{measured} \left( {t^{'} } \right) + \beta P_{cvp} } \right)dt^{'} + P_{measured} (0)} \right)$$

where *α* and *β* are *PWV/L*_*ao*_ and *1/RC*_*v*_ respectively.

### Parameter Identification

Parameter values *L*_*ao*_ and *β* were identified from the measured pressure waveform. In diastole, the measured pressure waveform decay can be assumed to result only from the release of energy stored by aortic compliance during systole. Thus, *P*_*measured*_ represents *P*_*res*_ in diastole, yielding:

11 $$P_{measured} \left( {td < t < tf} \right) \, = \, P_{res} \left( {td < t < tf} \right)$$

where *td* and *tf* are the time at the start of diastole and end of diastole, respectively.

**Fig. 4:**
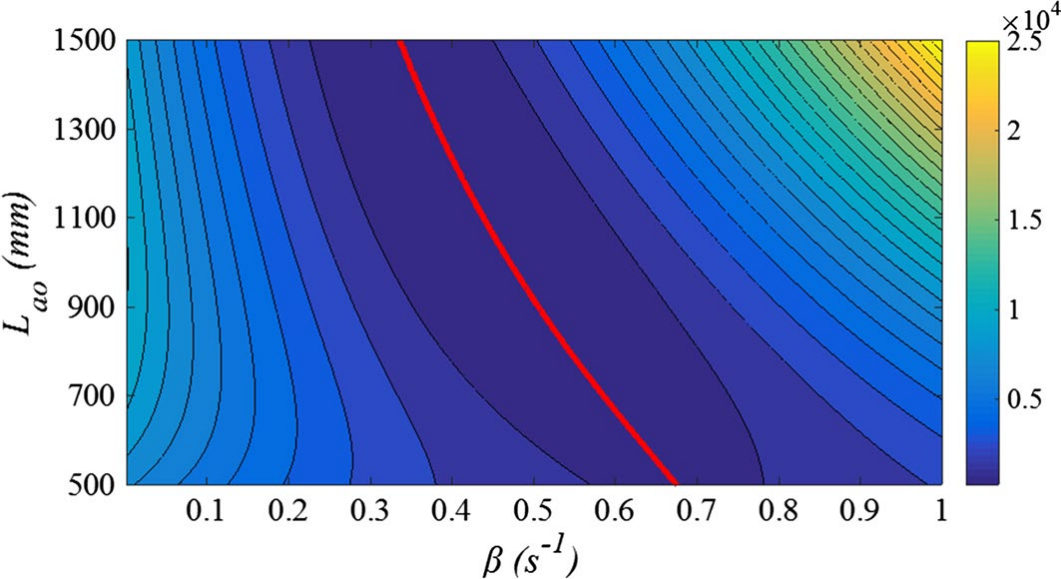
Error surface showing the discrepancy between *P*_*measured*_ *(td* < *t* < *tf)* and calculated *P*_*res*_ using different value of *L*_*ao*_ and *β* in Eq. 7. The *red line* represent optimal parameter *β* for a given *L*_*ao*_

By performing grid search for *L*_*ao*_ and *β*, the discrepancy between Eq. (10) and measured diastolic pressure decay was minimized. An example of the error surface produced from a grid search for a single beat is shown in Fig. 4. It can be seen in Fig. 4 that the surface is convex so an optimal value of *β* could be identified for each value of *L*_*ao*_.

**Fig. 5:**
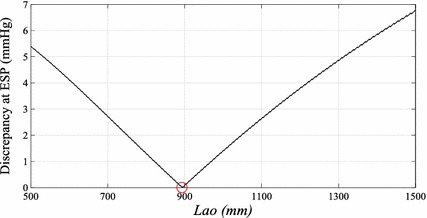
The* error curve* showing the discrepancy between *P*_*measured*_ at ESP and calculated *P*_*res*_ at ESP using optimal sets of *L*_*ao*_ and *β* identified by grid search. The *red circle* shows the identified optimal parameter *L*_*ao*_ for a given pressure waveform

To identify the most suitable set of parameters, *L*_*ao*_ and *β*, from the sets of optimal parameters identified by grid search, further constraints were added to the ESP to improve practical identifiability [27]. At end systole, ventricular hydraulic force equals aortic reservoir force pushing against the ventricle and thus, initiates closure of aortic valve. The flow entering into aorta from the ventricle would be zero at this point and, consequently, the excess pressure would be zero. Implementing this condition, the discrepancy between calculated end systolic pressure using the identified sets of *L*_*ao*_ and *β* and measured end systolic pressure were minimized to select an optimal set of parameters, *L*_*ao*_ and *β*, for a given pressure waveform. An example of error curve for this optimization process is shown in Fig. 5.

The parameter identification process described above was used for the first 10 beats of the experiment for each pig. Once 10 values of *L*_*ao*_ were identified for each pig, the values were averaged to give a representative *L*_*ao*_ for each pig over the rest of the study. To solve Eq. (10), *L*_*ao*_ was held constant because this anatomical length is not expected to change.

Using this fixed subject/pig-specific representative value of *L*_*ao*_, *β* was optimized using the condition defined in Eq. (11) for each heart beat and pressure waveform. The calculated *P*_*res*_ for each pressure waveform were then used to determine *P*_*ex*_ and, subsequently, used to calculate aortic flow and SV.

### Stroke volume estimation

The water hammer equation can be used to describe the relationship between PWV, changes in excess pressure (*dP*_*ex*_), and changes in velocity of blood through the aorta (*dU*_*ao*_) [28].

12 $$dP_{ex} = \, \rho PWVdU_{ao}$$

where *ρ* is the density of blood and assumed constant at 1050 kg/m^3^ [29]. Taking *Q*_*in*_ = *U*_*ao*_ *A*_*ao*_ and substituting Eq. (6) into (12), the aortic characteristic impedance, *Z*_*ao*_ can be expressed in terms of PWV, *ρ*, and *A*_*ao*_.

13 $$Z_{ao} = \, \rho PWV / A_{ao}$$

For this analysis, the first 10 beats of measured left ventricular SV values from the admittance catheter were used to calibrate *A*_*ao*_ for each pig. Identified *P*_*ex*_ and measured ${\text{SV}} = \mathop \smallint \limits_{0}^{{t_{d} }} Q_{in}$ were substituted into Eq. (6) to calculate *Z*_*ao*_. Using these *Z*_*ao*_ values, *A*_*ao*_ were determined from Eq. (13). The average value of *A*_*ao*_ for the first 10 beats was used to estimate SV.

### Relationship between model derived* A*_*ao*_ and systolic period

**Fig. 6:**
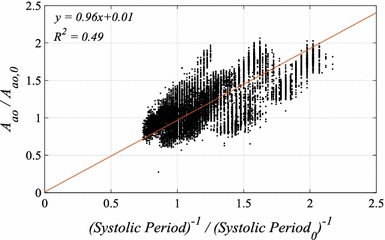
Correlation plot showing relationship between relative change in aortic area *A*_*ao*_ and systolic period relative to the calibration period. *A*_*ao,0*_ and *systolic period*_*0*_ represents the value obtained in the calibration period

To capture the relative change in values of *A*_*ao*_ over the course of an experiment, the relationship identified between *A*_*ao*_ calculated using the measured SV and systolic period identified from pressure waveform were used to improve the estimate of *A*_*ao*_. Figure 6 shows the regression line minimizing the geometric mean deviation between relative changes in these two parameters for all pigs in relation to calibrated point. Regression using geometric mean deviation produces larger error in the estimated *A*_*ao*_ from the relationship, however, this approach is more appropriate for this analysis as measurement error is expected in both parameters.

The relationship showed nearly 1:1 ratio in relative change between *A*_*ao*_ and systolic period. This relationship was applied to calibrated *A*_*ao*_ to identify a more accurate values of *Z*_*ao*_.

14 $$\frac{{A_{ao} }}{{A_{ao,0} }} = \frac{{Systolic \, Period_{0} }}{Systolic \, Period}$$

15 $$Z_{ao} = \frac{\rho PWV}{{A_{ao,0} }}\frac{ Systolic \, Period}{{Systolic \, Period_{0} }}$$

where the subscript zero refers to calibration period, where *A*_*ao*_ was obtained using 10 heart beats.

### Stroke volume

The calculated aortic characteristic impedance from Eq. (15) and *P*_*ex*_ using Eqs. (3) and (10) were then used to estimate SV.

16 $$SV_{estimate} = \frac{1}{{Z_{ao} }}\mathop \int \limits_{0}^{tf} P_{ex} (t)dt$$

The SV values estimated using Eq. (16) are compared against measured SV from the admittance catheter using Bland–Altman plots. The schematic of processes involved in the pressure contour method are outlined in Appendix.

## Results

**Fig. 7:**
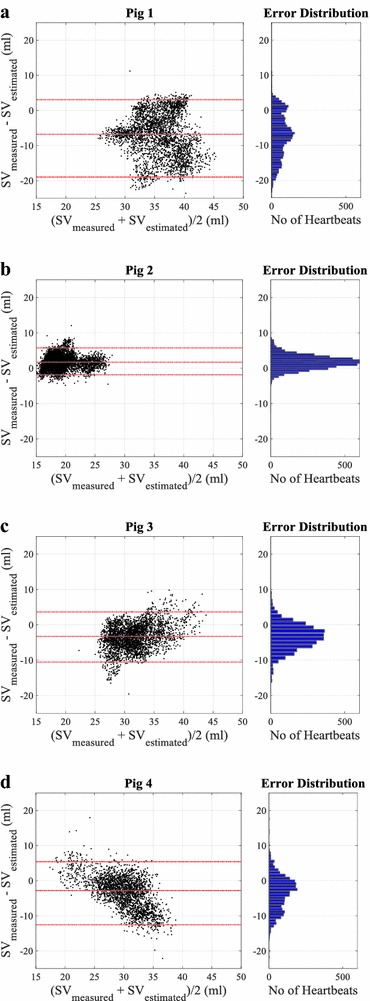
Bland–Altman plots **a**–**d** showing agreements between measured and estimated SV for all pigs. *Red dashed line* showing the bias and 95% interval. *Right panel* showing the error distribution between measured and estimated SV values

**Fig. 8:**
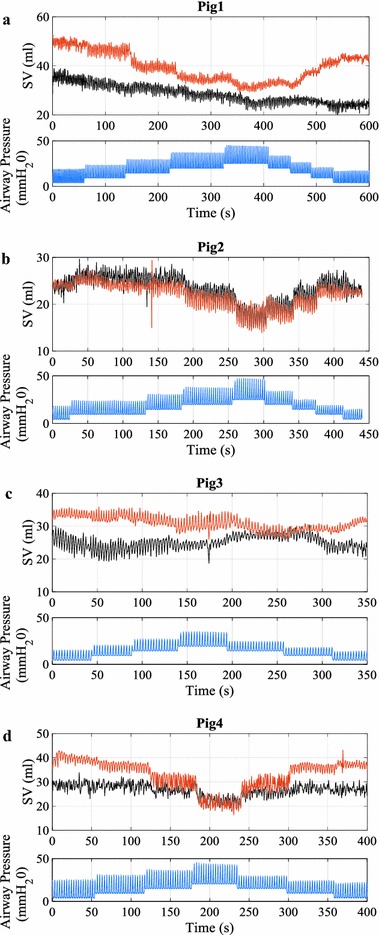
Time series plot **a**–**d** showing measured and estimated SV in the last RM period of the experiment for all pigs. *Top panel* measured SV from admittance catheter (*black line*) and estimated SV using Eq. (13) (*red line*). *Bottom panel* simultaneously measured airway pressure showing PEEP changes during recruitment manoeuvers RM (*blue line*)

The identified values of *L*_*ao*_, *A*_*ao,0*_, relevant physiological parameters, total number of heart beats analysed, and weight for each pig are shown in Table 2. Bland–Altman plots comparing SV estimated using Eq. (16) and SV measured from the admittance catheter are presented in Fig. 7. The summary of bias, 95% interval, and precision calculated as half of 95% range divided by mean SV for each pig are shown in Table 3. In addition, time series showing measured and estimated SV in the last RM period, the most distant point from calibration and thus a potential worst case, for each pig are shown in Fig. 8.

**Table 2:** Summary of identified parameters *L*_*ao*_, *A*_*ao,0*_ for all pigs and ranges of physiological parameters, mean aortic pressure (MAP), PWV, systolic period, and measured SV for volume expansion and dobutamine period

	Pig 1	Pig 2	Pig 3	Pig 4
Weight (kg)				
	24.5	20	23.5	23.3
Identified aortic dimension				
L-_ao_ (m)	0.88	0.41	0.89	0.91
A_ao,0_ (mm^2^)	201	269	473	163
Volume expansion				
MAP (mmHg)	152 [136 to 182]	114 [93 to 136]	111 [93 to 132]	55 [42 to 77]
PWV (m/s)	5.5 [5.3 to 6.0]	6.7 [6.4 to 6.9]	8.5 [8.2 to 8.9]	3.9 [3.7 to 4.1]
Heart rate (beats/min)	64 [62 to 67]	86 [78 to 94]	81 [73 to 88]	71 [69 to 74]
Systolic period (s)	0.38 [0.33 to 0.46]	0.32 [0.25 to 0.39]	0.31 [0.26 to 0.38]	0.33 [0.31 to 0.36]
SV_measured_ (ml)	34 [28 to 40]	19 [16 to 23]	30 [26 to 39]	29 [25 to 34]
Dobutamine/+ volume expansion				
Pressure (mmHg)	150 [123 to 184]	90 [54 to 124]	100 [79 to 123]	61 [43 to 95]
PWV (m/s)	6.0 [5.6 to 6.7]	6.3 [5.4 to 6.8]	8.2 [7.6 to 8.8]	4.6 [4.3 to 5.2]
Heart rate (beats/min)	104 [90 to 108]	141 [136 to 142]	109 [98 to 120]	107 [93 to 115]
Systolic period (s)	0.23 [0.22 to 0.31]	0.19 [0.18 to 0.22]	0.23 [0.19 to 0.3]	0.23 [0.21 to 0.31]
SV_meansreud_ (ml)	28 [23 to 36]	24 [17 to 27]	25 [21 to 28]	27 [22 to 31]
Total no of heart beats analysed				
	2956	4945	3057	2451

Data are presented as the mean [5th–95th percentiles]

**Table 3:** Summary of Bland–Altman analysis for each pigs

Pig no.	Bland–Altman results (ml)	Precision (%)
Pig 1	−6.8 [−18.9 to 3.0]	31
Pig 2	1.7 [−1.8 to 5.7]	20
Pig 3	−3.3 [−10.5 to 3.6]	22
Pig 4	−2.8 [−12.5 to 5.4]	30

Data are presented as the bias [2.5th–97.5th percentiles], where bias is the mean difference between measured and estimated SV. Precision is calculated as half of 95% range divided by mean SV for each pig

## Discussion

To investigate the accuracy of the method, wide ranges of physiological conditions were analysed involving significant changes in heart rate, preload, afterload, and contractility of the heart. Fluid state and airway pressure were altered to induce preload changes producing more than 30 percent difference in SV. Dobutamine was infused to induce afterload and contractility changes involving decrease in ventricular-arterial coupling [30]. In addition, dobutamine increased heart rate by 50% in all pigs as can be seen in Table 2.

The Bland–Altman results in Fig. 7 and Table 3 demonstrate the ability of the method in capturing the absolute value of SV. Despite all the significant hemodynamic changes, 95% range was within ±10 ml (approximately ±30%) for all pigs and many errors were much smaller. The method showed it is capable of estimating clinically acceptable absolute SV values according to Critchely and Critchely [31], where they states acceptable accuracy of the stroke volume estimation method against the reference method to be within approximately ±30%.

The ability of the method to capture SV trend/dynamics is shown in Fig. 8. The figure shows the last RM period of the experiment where one would expect to find the largest difference in the hemodynamic conditions. It can be seen that PEEP induced stroke volume trends were correctly captured in most cases. The method was able to capture both ‘affected’ (Pig 1, 2, and 4) and ‘unaffected’ (Pig 3) cases, which has clinical importance in preload assessment [32]. In addition, Stroke Volume Variability (SVV) induced from individual breath (cyclic effect) was correctly captured.

It can be noted that the method over-estimated the trend for Pig 4 and made incorrect estimation for Pig 1 during PEEP reduction. Pig 4 had an extremely low blood pressure, of approximately 45 mmHg at the beginning of the experiment where the method was calibrated and had the biggest change in the mean pressure during the experiment, as can be seen in Table 2 (almost a twofold increase). In such a case, the method was not able to capture the dramatic change in aortic dimension and therefore, deviated from true value of SV. The error seen in Pig 1 was not able to be resolved as pressure and PWV values returned to normal after RM, similar to starting point of RM. However, the value of SV remained at a reduced value. In both cases, further investigations are necessary and there are potential to further improve the method.

### Pressure contour analysis

There are several methods currently available for estimating continuous SV by pressure contour analysis [33]. In general, the Windkessels models are used and parameters involved in the model are identified from the single pressure waveform and/or patients’ demographic/physical characteristics [34]. The significant improvement of the presented method over conventional methods is that PWV measurements are integrated into pressure contour analysis to accurately capture the dynamics of time-varying model parameters, which are needed to obtain SV correctly.

Pulse wave velocity measurements are related to arterial distensibility [35] and its relative changes within a subject provide additional information on the cause of changes in the characteristics of the pressure waveform. A combination of changes in the value of PWV and changes in the shape of diastolic pressure decay for a given aortic dimension gives a better approximation of the time-varying arterial reservoir function. The reservoir pressure waveform estimates the minimum pressure that ventricle must provide to induce flow into the artery [36] and is closely related to arterial impedance [37]. By assigning the correct components of the pressure waveform to each of the Windkessel parameters, and with added data inputs, the method is capable of capturing accurate physiological conditions and its changes.

### Estimation of aortic dimension

In this work, the aortic dimension was estimated by assuming the aorta behaved as a simple cylindrical tube having uniform properties and pressure along it. In reality, the properties are non-uniform and each segment of aorta has different pressure contours. However, to obtain such a large amount of information in a clinical settings is impractical, if not impossible. These assumptions thus produce error in the estimation of SV, but are necessary in developing a method based on clinically accessible and reasonable measurements.

The identified values of *L*_*ao*_ and calibrated values of *A*_*ao*_ for each pig are shown in Table 2. These values represent an estimate of aortic dimensions for a given value of pressure contour and PWV. It is largely affected by the absolute value of PWV and SV values used to calibrate the method. Since even the reference values are expected to have error of approximately ±20% [38], the identified values of aortic dimension may not be entirely realistic. However, given the large errors of reference values, the resulting values are of use, as are the resulting SV values.

The important part of this calibration is to give an estimate of the dimension under given conditions and to use the relative changes in the measurements to track the trends of hemodynamic parameters. In a clinical environment, the absolute value of SV is not of great importance. However, the relative changes in SV for different patient conditions and in response to therapy is of major clinical significance [39]. From this point of view, the method has shown the ability to sufficiently track the trends of SV (Fig. 8) under the assumptions made.

### Relationship between model derived* A*_*ao*_ and systolic period

The identified relationship shown in Fig. 6 uses *A*_*ao*_ derived from Eq. (13) using the measured value of SV. In the experiment, true aortic diameter was not measured and consequently, the exact physiological reason behind the relationship between aortic area and systolic period was not able to be determined. This limitation is due to the possibility that this relationship may represent correlation between systolic periods with other physiological variables.

Previous studies have identified the relationship between heart rate/ejection time and aortic PWV [40, 41]. The relationship can be explained by stiffening of the aortic wall due to viscoelastic properties. Equation (13), which is derived from the water hammer equation, fails to describe the energy loss due to hysteresis of the aorta and could thus have produced incorrect values of *A*_*ao*_.

To identify the exact source of the relationship presented in Fig. 6, accurate measurements of time-varying aortic area are required. Such measurements will produce a stress–strain relationship explaining the time-dependent mechanisms associated with arterial distensibility/stiffness. However, whether the relationship is due to the viscoelastic properties or not, the identified population relationship is still useful in providing an accurate estimation of SV and thus, it is used for the method presented until better data is available in subsequent experiments.

### Extending the method to alternative PWV estimation

The experiments used here involved highly invasive method to obtain PWV, requiring two pressure measurements along aorta, which is uncommon in the critical care environment. However, the results from this analysis demonstrates the possibilities for more accurate estimation of SV if PWV can be estimated by this or different approach. The aortic arch pressure measurement was used only for the purpose of identifying the transit time and the information from its contour was not used. Therefore, this measurement can potentially be replaced with less invasive measurements such as ECG to detect another pulse location [42]. Previous studies show evidence for strong relationship between invasive and non-invasive PWV measurements [43, 44], and thus the method is expected to have negligible influence from use of alternative PWV measurements.

### Limitations

The clinical applicability of the method presented is limited to the patient having at least one pressure measurement from a catheter in a central artery. Pressure sensing lumens in radial artery are commonly used in ICU patients. However, due to the current perceived risk-to-benefit ratio for having central pressure waveform, it is rarely used in clinical practice [45]. The ability and improved accuracy of estimating SV provided by the method may reverse this trend turning potential risk into benefit [46, 47]. Furthermore, identified time-varying Windkessel parameters (aortic characteristic impedance/compliance and systemic resistance) provides further insight into optimization of clinical treatment [48].

Another limitation of this study is that experiments were performed on healthy pigs and more complicated situations involving valve regurgitation, aneurysm, and/or any arterial defect were not analysed. Irregular pressure contours produced by these conditions could affect the parameter identification process and precision. To investigate the clinical applicability of the method in such cases, further validation and analysis must be done covering wider range of cardiovascular systems and circulatory dysfunctions.

## Conclusion

The method presented in this paper accurately estimates and tracks SV trends even when hemodynamic properties are significantly altered. PWV measurements, which are usually available in the ICU or can easily be obtained non-invasively, were integrated into pressure contour analysis to overcome weaknesses associated with conventional methods for estimating SV. The additional information gained by PWV allowed precise estimation of Windkessel parameters and thus, accurate estimation of SV. The Bland–Altman plots showed average 95% limits of agreement of ±26% between estimated SV and the reference SV across all pigs, demonstrating the clinical applicability of the method. In addition, the method require only one calibration per subject making the method more practical. The method presented can track accurate and clinically important changes in patients’ haemodynamic state providing essential information for correct diagnosis and optimal care.

## Figures and Tables

**Fig. 9:**
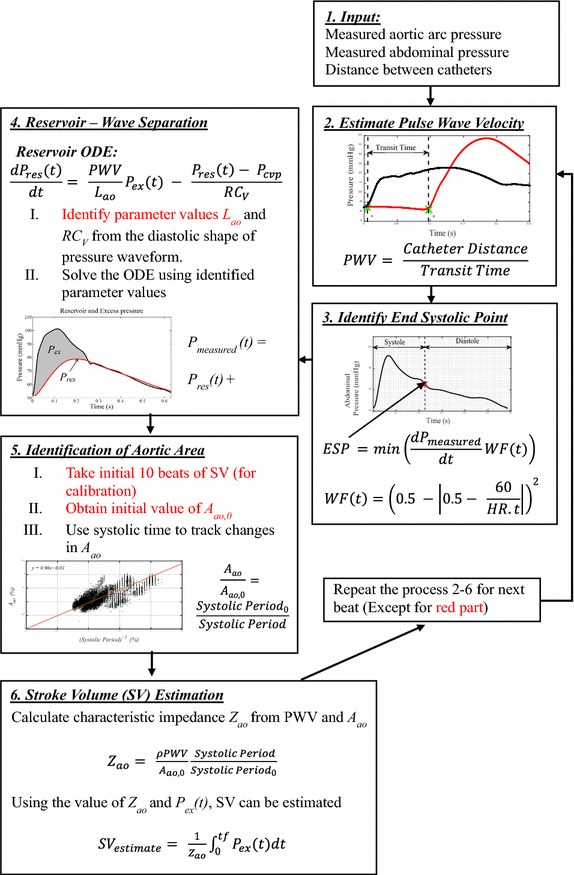
Schematic of SV estimation processes showing key steps involved in the pressure contour method

## Appendix

See Fig. 9.
